# Health effects of a forest environment on natural killer cells in humans: an observational pilot study

**DOI:** 10.18632/oncotarget.24741

**Published:** 2018-03-27

**Authors:** Tsung-Ming Tsao, Ming-Jer Tsai, Jing-Shiang Hwang, Wen-Fang Cheng, Chang-Fu Wu, Charles-C.K. Chou, Ta-Chen Su

**Affiliations:** ^1^ The Experimental Forest, National Taiwan University, Nantou, Taiwan; ^2^ School of Forestry and Resource Conservation, National Taiwan University, Taipei, Taiwan; ^3^ Institute of Statistical Science, Academia Sinica, Taipei, Taiwan; ^4^ Department of Obstetrics and Gynecology, National Taiwan University Hospital, Taipei, Taiwan; ^5^ Institute of Occupational Medicine and Industrial Hygiene, College of Public Health, National Taiwan University, Taipei, Taiwan; ^6^ Research Center for Environmental Changes, Academia Sinica, Taipei, Taiwan; ^7^ Department of Internal Medicine and Cardiovascular Center, National Taiwan University Hospital, Taipei, Taiwan

**Keywords:** natural killer cells, activating NK cells, forest trip, forest environment, urban environment

## Abstract

Health effect assessments based on natural killer (NK) cells are an important emerging area of human health. We recruited 90 forest staff members in Xitou, Taiwan and 110 urban staff members in Taipei to investigate the health effects of forest environment exposure on NK cells (CD3^−^/CD56^+^) and activating NK cells (CD3^−^/CD56^+^/CD69^+^) in humans. We also invited 11 middle-aged volunteers in a pilot study to participate in a five-day/four-night forest trip to Xitou forest to investigate the health effects of a forest trip on NK cells and activating NK cells. Results showed that NK cells were higher in the forest group (19.5 ± 9.1%) than in the urban group (16.4 ± 8.4%). In particular, the percentage of NK cells was significantly higher in the forest group than in the urban group among the subgroups of male, a higher body mass index (≥ 25 kg/m^2^), without hypertension, lower high-sensitivity C-reactive protein, hyperglycemia, without smoking habit, and with tea drinking habit. After the five-day trip in Xitou forest, the percentage of activating NK cells of the invited participants from Taipei increased significantly after the trip to Xitou forest (0.83 ± 0.39% vs. 1.72 ± 0.1%). The percentage of activating NK cells was 1.13 ± 0.43%, which was higher than the baseline value of 0.77 ± 0.38% before the forest trip among the seven subjects who participated in the follow-up study four days after returning to Taipei. This study suggests that exposure to forest environments might enhance the immune response of NK cells and activating NK cells in humans.

## INTRODUCTION

Natural killer (NK) cells are a subset of lymphocytes with a distinct morphology and the ability to directly kill certain target cells via one or more cytolytic mechanisms. They are important in the human endocrine and immune systems to induce tumor or virus-infected targeted cell death [1, 2]. Activating NK cells produce anti-cancer proteins, such as perforin and granzyme, in host anti-cancer defense mechanisms, where the targeting of cancer cells is known to be of critical importance [2]. Among the cytolytic effector lymphocytes in the innate immune system, NK cells are especially critical for immune surveillance of tumors [2–4]. Studies have shown that immunosuppression is common after standard anti-cancer treatments and that NK cells are particularly suppressed. For example, peripheral blood natural cytotoxicity is diminished significantly in breast cancer patients [3–5]. Human immune systems are highly diverse and not completely understood at present, but recent studies indicate that environmental and non-heritable factors make much larger contributions than heritable factors [6, 7]. Considerable evidence indicates that forest environments can enhance the percentage and function of NK cells and intracellular anti-cancer proteins in lymphocytes [8–12]. Thus, exposure to a forest environment can have health-promoting effects.

Forest environments may have some positive health effects on physiological and psychological activities. In particular, the beneficial physiological health effects of a forest environment enhance immune functions, including human NK cells [8–10], decrease sympathetic nerve activity, enhance parasympathetic nerve activity, and lower blood pressure, pulse rate, heart rate variability, and cholesterol concentration [13–20]. Forest environments are more greatly associated with positive health effects on subclinical cardiovascular disease and health-related quality of life compared with urban environments [21]. Exposure to a forest environment significantly suppressed sympathetic activity and increased parasympathetic activity in young Japanese male adults who participated in a three-day/two-night field experiment compared with an urban environment according to a heart rate variability analysis [14]. Phytoncides from wood essential oils, such as α-pinene and limonene, have been shown to induce human NK cells, as well as the expression levels of perforin, granzyme A, and granulysin [22]. Studies have reported that a forest trip enhanced human NK cells and intracellular anti-cancer protein levels in lymphocytes; moreover, the increased NK cell activity lasted for more than seven days after the forest trip [8–11]. However, these previous studies investigated only the experimental effects of a short-term forest trip on human NK cells. More studies are required to evaluate the health effects of exposure to a forest environment on human NK cells.

The objectives of this pilot study were the following: (1) to determine the percentage of NK cells (CD3^−^/CD56^+^) and activating NK cells (CD3^−^/CD56^+^/CD69^+^) in people who live in a forest environment compared with those who live in an urban environment; and (2) to determine the health effects of a forest trip on NK cells and activating NK cells in participants who stayed for five days and four nights in a forest environment.

## RESULTS

The mean ages in the urban and forest groups were 44.8 and 45.2 years, respectively (Table 1). The forest group contained more male participants (66.7%) than the urban group (53.6%). The systolic and diastolic blood pressure readings in the forest group were higher (130.3 and 83.2 mmHg, respectively) than in the urban group. Lifestyle habits in terms of alcohol consumption, smoking, coffee, and tea differed significantly among the two groups. In particular, habitual tea drinking was higher in the forest group (87.8%) than in the urban group (62.7%). However, habitual coffee consumption was higher in the urban group (70.9%) than in the forest group (53.3%).

**Table 1:** General characteristics of staff members living in urban and forest environments

	Environment		
	Urban	Forest	
	*N* = 110	*N* = 90	*p*-value^d^
Age (year)	44.8 ± 6.6	45.2 ± 10.6	0.704
Male sex (%)	53.64	66.67	0.062
Body mass index (kg/m^2^)	23.8 ± 3.48	24.59 ± 3.54	0.113
Waist (cm)	81.52 ± 9.61	84.15 ± 8.80	0.049
Systolic blood pressure (mmHg)	125.06 ± 14.01	130.34 ± 15.28	0.012
Diastolic blood pressure (mmHg)	80.07 ± 10.75	83.19 ± 10.17	0.039
Hypertension (%)	26.36	24.44	0.757
Hypertension with medication (%)	9.09	10.0	0.827
Fasting glucose (mg/dL)	85.83 ± 9.28	91.60 ± 10.92	< .001
Cholesterol (mg/dL)	188.84 ± 35.25	191.87 ± 38.69	0.563
Triglycerides (mg/dL)	106.40 ± 52.21	122.34 ± 85.22	0.123
HDL-cholesterol (mg/dL)	56.57 ± 14.65	54.81 ± 11.78	0.347
LDL-cholesterol (mg/dL)	113.08 ± 30.19	118.30 ± 34.71	0.257
hs-CRP (mg/dL)	0.14 ± 0.18	0.21 ± 0.45	0.169
Alcohol drinking ^a^ (%)	18.18	36.67	0.003
Smoking habit ^b^ (%)	20.91	34.44	0.032
Exercise habit ^c^ (%)	54.72	59.55	0.497
Coffee (%)	70.91	53.33	0.010
Tea (%)	62.73	87.78	< .001

HDL and LDL cholesterol: high-density and low-density lipoprotein cholesterol; hs-CRP: high-sensitivity C-reactive protein; ^a^drinking habit: drinking alcohol once or more per week; ^b^smoking habit: current smoker and ex-smoker; ^c^exercise habit: three times with at least 30 min each per week; ^d^Continuous variables were expressed as mean ± SD, and *t*-test was used to perform comparisons.

The mean environmental monitoring results are presented in Table 2, including air pollutants, temperature, and relative humidity. The SO_2_, NO, NO_2_, NO_x_, CO, and temperature levels were significantly lower in the forest environment compared with those in Taipei (indoors and outdoors). However, the levels of O_3_ and relative humidity were higher in Xitou than those in Taipei. The complete blood cell counts are presented in Table 3. The red blood cell, hemoglobin, hematocrit, and white blood cell counts (e.g., lymphocyte and monocyte) were significantly higher in the forest group than those in the urban group.

**Table 2:** Comparisons of the air quality and meteorological data in the forest and urban environments

	Forest	Urban		*P*_*1*_ value	*P*_*2*_ value
	^a^*N* = 96	Indoor *N = 94*	outdoor *N = 96*		
SO_2_ (ppb)	0.45 ± 0.46	0.17 ± 0.18	3.04 ± 1.43	< .001	< .001
NO (ppb)	0.27 ± 0.24	39.22 ± 22.97	12.45 ± 11.6	< .001	< .001
NO_2_ (ppb)	2.61 ± 2.19	4.45 ± 1.61	30.34 ± 9.02	< .001	< .001
NO_x_ (ppb)	2.44 ± 2.17	43.67 ± 22.14	42.79 ± 18.4	< .001	< .001
CO (ppm)	0.37 ± 0.19	1.09 ± 0.51	0.87 ± 0.31	< .001	< .001
O_3_ (ppb)	27.48 ± 16.15	6.65 ± 0.73	13.56 ± 10.4	< .001	< .001
Temperature (°C)	13.05 ± 1.91	17.94 ± 1.03	17.35 ± 1.69	< .001	< .001
Relative humidity	91.36 ± 5.60	67.86 ± 2.86	82.33 ± 6.45	< .001	< .001

*P*_*1*_ value corresponds to *t*-test comparisons of the Xitou and urban sites (indoor). *P*_*2*_ value corresponds to *t*-test comparisons of the Xitou and Wanhua sites (outdoor) of the Environmental Protection Agency, Taipei, Taiwan. ^a^*N* corresponds to the sample size for the hourly average data.

**Table 3:** Comparisons of complete blood cell counts for the urban and forest groups

	Urban	Forest	
	*N* = 110	*N* = 90	*p*-value^a^
Red blood cell (10^6^/μL)	4.97 ± 0.61	5.18 ± 0.60	0.014
Hemoglobin (g/dL)	14.18 ± 1.83	15.03 ± 1.57	0.001
Hematocrit (%)	42.4 ± 4.48	44.19 ± 3.89	0.003
White blood cell (10^3^/μL)	5.76 ± 1.43	6.29 ± 1.54	0.013
Neutrophil (10^3^/μL)	3.36 ± 1.05	3.63 ± 1.20	0.093
Lymphocyte (10^3^/μL)	1.93 ± 0.56	2.10 ± 0.59	0.034
Monocyte (10^3^/μL)	0.30 ± 0.11	0.35 ± 0.11	0.005
Eosinophil (10^3^/μL)	0.13 ± 0.10	0.16 ± 0.11	0.066
Basophil (10^3^/μL)	0.03 ± 0.02	0.03 ± 0.02	0.439
Platelet (10^3^/μL)	258.17 ± 51.62	251.48 ± 47.41	0.345

^a^Continuous variables were expressed as mean ± SD, and *t*-tests were used to perform comparisons.

Table 4 shows that the percentage of NK cells was positively correlated with male, age, systolic and diastolic blood pressure, high-sensitivity C-reactive protein, and fasting glucose levels. However, the percentage of activating NK cells was not correlated with all cardiovascular characteristics.

**Table 4:** Correlation coefficients between NK cell, activating NK cell, and cardiovascular characteristics among staff members living in urban and forest environments (*N* = 200)

	NK cell		Activating NK cell	
	Correlation coefficient	*P*-value	Correlation coefficient	*P*-value
Male	0.192	0.007	0.056	0.428
Age	0.260	< .001	–0.011	0.879
Body mass index	0.073	0.308	0.128	0.072
Systolic blood pressure	0.315	< .001	0.056	0.435
Diastolic blood pressure	0.353	< .001	0.081	0.256
Triglycerides	0.135	0.057	0.062	0.385
Cholesterol	0.123	0.083	–0.092	0.194
High-sensitivity C-reactive protein	0.144	0.042	0.034	0.631
Fasting glucose	0.174	0.014	–0.051	0.475
Smoking	0.057	0.424	0.054	0.450
Alcohol	0.126	0.075	–0.038	0.594
Coffee	–0.136	0.056	0.049	0.487
Tea	0.064	0.366	0.027	0.697

Table 5 shows that the percentage of NK cells was higher in the forest group (19.5 ± 9.1%) than in the urban group (16.4 ± 8.4%). After adjusting for age and gender, the regression estimates indicated that the percentage of NK cells, but not activating NK cells, were significantly higher in the forest group than in the urban group. Subgroup analyses for NK cells differences between two groups are also shown in Table 5. The percentage of NK cells was significantly higher in the forest group than in the urban group among subgroups of male, higher BMI (≥ 25 kg/m^2^), without hypertension, lower hs-CRP levels, hyperglycemia (fasting glucose ≥ 100 mg/dL), without smoking habit, and with tea drinking habit. However, all subgroups did not differ significantly in terms of the percentage of activating NK cells between the two groups.

**Table 5:** Comparisons of NK cell and activating NK cell between staff members living in urban and forest environments

				NK cell (%)							Activating NK cell (%)					
				Urban			Forest		*P*		Urban		Forest		*P*	
		*N*		*N* = 110			*N* = 90		value		*N* = 110		*N* = 90		value	
Crude value				16.4 ± 8.4			19.5 ± 9.1		0.01		1.81 ± 2.85		1.55 ± 1.14		0.37	
Adjusted value^a^				16.7 ± 0.8			19.2 ± 0.9		0.03		1.83 ± 0.22		1.53 ± 0.24		0.35	
Age, years																
< 50	143			15.9 ± 7.7			17.4 ± 8.1		0.27		1.96 ± 3.17		1.5 ± 1.03		0.22	
≥ 50	57			18.4 ± 10.7			23.0 ± 9.7		0.10		1.28 ± 0.73		1.63 ± 1.31		0.21	
Gender																
Female	81			15.2 ± 8.5			16.7 ± 8.3		0.44		1.52 ± 2.47		1.57 ± 1.51		0.91	
Male	119			17.5 ± 8.3			20.9 ± 9.2		0.04		2.07 ± 3.14		1.54 ± 0.92		0.22	
Body mass index (g/m^2^)																
< 25		131		16.4 ± 8.4			18.0 ± 8.6		0.29		1.63 ± 2.20		1.58 ± 1.27		0.88	
≥ 25		69		16.5 ± 8.7			21.6 ± 9.4		0.02		2.29 ± 4.07		1.50 ± 0.95		0.30	
Hypertension (%)																
No		149		15.6 ± 7.5			18.4 ± 8.8		0.04		1.74 ± 2.72		1.57 ± 1.19		0.61	
Yes		51		18.8 ± 10.3			23.1 ± 9.2		0.13		2.02 ± 3.23		1.48 ± 1.01		0.41	
High-sensitivity C-reactive protein (mg/dL)																
< 50th		100		14.7 ± 6.9			19.1 ± 9.0		0.01		1.33 ± 1.05		1.48 ± 0.88		0.44	
≥ 50th		100		18.5 ± 9.6			19.8 ± 9.2		0.48		2.40 ± 4.01		1.60 ± 1.32		0.19	
Fasting glucose (mg/dL)																
< 100		167			16.8 ± 8.7		19.1 ± 9.4		0.11		1.87 ± 3.0		1.49 ± 1.14		0.26	
≥ 100		33			13.4 ± 5.5		20.9 ± 7.9		0.01		1.38 ± 0.97		1.74 ± 1.14		0.37	
Smoking habit																
Yes			146			17.2 ± 10.3		19.7 ± 9.0		0.33		2.57 ± 4.67		1.39 ± 0.77		0.24
No			54			16.2 ± 7.9		19.4 ± 9.2		0.03		1.61 ± 1.16		1.63 ± 1.29		0.95
Alcohol habit																
Yes			53			17.2 ± 8.7		21.2 ± 8.4		0.10		1.67 ± 1.07		1.48 ± 0.93		0.52
No			147			16.3 ± 8.4		18.5 ± 9.4		0.13		1.85 ± 3.11		1.58 ± 1.25		0.48
Coffee																
Yes			126			16.0 ± 8.4		18.3 ± 8.4		0.13		1.88 ± 3.32		1.62 ± 1.17		0.52
No			74			17.4 ± 8.4		20.9 ± 9.8		0.12		16.5 ± 10.7		14.7 ± 11.2		0.48
Tea																
Yes			148			16.4 ± 8.0		19.7 ± 8.6		0.02		1.98 ± 3.49		1.51 ± 1.0		0.28
No			52			16.5 ± 9.1		18.1 ± 12.4		0.64		15.3 ± 11.5		18.2 ± 19.0		0.63

^a^ Regression model estimates adjusting for age and gender, and data are expressed as mean ± standard deviation in subgroup analyses.

Table 6 shows that among the 11 participants, 27.3% were male and the average age was 60.4 years. The participants from Taipei were invited to attend a forest trip (five-day/four-night) to the Xitou Nature Education Area from January 6 to 10, 2014. No event of skin wound or bee bites was reported by the participants during the forest trip. Compared with the measurements obtained in Taipei one day before the forest trip, the percentage of activating NK cells increased significantly among the 11 participants after the forest trip (*p* = 0.002). However, no significant difference in their percentage of NK cells was observed after the forest trip, as shown in Table 6A. Although the percentage of activating NK cells in the participants decreased after the participants left the forest, the forest trip significantly increased the percentage of activating NK cells compared with pre-forest trip levels. This change lasted for at least four days, as shown in Table 6B. The percentage of activating NK cells was 1.13 ± 0.43%, which was higher than the baseline value of 0.77 ± 0.38% before the forest trip among the seven subjects who completed in the follow-up study.

**Table 6:** Effects of exposure to a forest environment on NK cell and activating NK cell

(A) Comparisons before and after the forest trip in Xitou for five days					
*N* = 11	Pre-forest trip		Post-forest trip	Paired-difference	*p*-value^a^
NK cell level (%)		22.49 ± 11.76	23.72 ± 15.05	1.23	0.411
Activating NK cell (%)		0.83 ± 0.39	1.72 ± 0.1	0.89	0.002

(B) Comparisons before and after the forest trip and four days after returning to Taipei				
*N* = 7	Pre-forest trip	Four days after	Paired-difference	*p*-value
NK cell level (%)	20.51 ± 11.27	20.98 ± 10.99	0.47	0.746
Activating NK cell (%)	0.77 ± 0.38	1.13 ± 0.43	0.36	0.004

^a^*P*-value by using paired *t*-tests.

Participant characteristics: data was shown in average.

(A) group: age_,_ 60.4 years; body mass index_,_ 23.54 kg/m^2^; male, 27.3%; high-sensitivity C-reactive protein_,_ 0.12 mg/L; systolic blood pressure_,_ 113 mmHg.

(B) group: age_,_ 61.8 years; body mass index_,_ 23.57 kg/m^2^; male, 14.3%; high-sensitivity C-reactive protein_,_ 0.13 mg/L; systolic blood pressure_,_ 106 mmHg.

## DISCUSSION

This study is the first to demonstrate the health effects of a forest environment on human NK cells by comparing subjects who live in a forest environment with those who live in an urban environment. The effects were more significant in subjects of overweight, male, and with hyperglycemia. This finding indicates that the immune response of people with specific cardiovascular risk factors may be improved by living in a forest environment. In this study, we further demonstrated the health effects of a forest trip on immune function in terms of activating NK cells. For health effects of a forest environment on natural killer cells, studies in which 13 healthy nurses and 12 healthy males took a three-day/two-night forest trip showed that the increased NK cell activation could last for more than seven days after the forest trip [9, 10]. Our finding is consistent with Li’s finding [9, 10] that a forest trip can enhance immune response in terms of activating NK cells and the effects can last for more than four days.

Maintaining enough NK cells is essential for healthy aging. In general, the NK cells increased with age [23]. Our results showed that study subjects in the forest trip group had a higher NK cell percentage than the forest and urban groups, which might be due to the older age of the subjects of the forest trip group than the other two groups (60 vs. 45 years). NK cells (CD56^+^) are divided into CD56^bright^ and CD56^dim^ major subsets that have both different receptor profiles and functions [24]. Lutz et al. [25] reported that NK (CD56^bright^) cells proliferated rapidly but died relatively slowly. Aging has differential effects on NK (CD56^bright^) and NK (CD56^dim^) cell subsets. Older individuals presented with a significantly higher percentage of NK (CD56^dim^) cells but a significantly lower proportion of NK (CD56^bright^) cells, thus resulting in an increased NK (CD56^dim^) to NK (CD56^bright^) ratio [25, 26]. The age-related increase in the NK cell percentage in older adults may be the result of an accumulation of long-lived NK cells [27].

NK cells are important in the human endocrine and immune systems as first-line effectors to induce tumor or virus-infected targeted cell death [1–2]. Activating NK cells have the ability to directly kill certain target cells. Many studies have demonstrated that NK cell subset distribution and the change in the expression of early activation antigen, CD3^−^/CD56^+^/CD69^+^ NK cells with respect to clinical response [5, 28–30]. Although the activating NK cells have been established to have the ability to attack the target cells, we still have to actually carry out the activity assay of killing target cells for understanding NK cell activity. In general, NK cell activity was assayed according to the standard microtiter ^51^Cr-release assay [28, 31]. Consistent evidence from both epidemiological and experimental studies have demonstrated that alcohol consumption [32–33], physical exercise [34], circadian variation [35], menstruation [36], cancer [37–38], age [26, 39], smoking habits [40–41], and environment [10] can affect NK cell activity in human. Furthermore, many studies have demonstrated that a forest environment can enhance the immune response as measured by NK cell activity, and the percentage and absolute numbers of NK cells [8–10].

NK cells in humans are dependent on environmental factors, alcohol consumption, physical exercise, circadian variation, and food [28, 31, 35, 42–43]. Previous studies also suggest that exposure to phytoncides and decreased stress hormone levels may have partially contributed to the increases in human NK cells after the forest trip. In particular, both the environmental factors related to the forest and cardiovascular characteristics were the major factors that affected human NK cells. The specific features of the Xitou forest comprise the biogenic volatile organic compounds emitted by the leaves of *Cryptomeria japonica* trees, including phytoncides such as α-pinene, limonene, and cedrol [44]. A previous study in Japan showed that subjects with healthy lifestyles, such as nonsmokers and those who exercised regularly, had significantly higher NK cells and more perforin, granulysin, and granzyme A/B-expressing cells than subjects with poor lifestyles [45]. Effects of phytoncides from wood essential oils significantly increased NK cell, and perforin, granulysin, and granzyme A/B-expressing cells, whereas they significantly decreased the percentage of T cells, thereby indicating that phytoncide exposure may affect human immune function [46].

We also measured the phytoncides and negative ions during outdoor monitoring of forest and urban environments and indoor monitoring of a wooden house made of Western red cedar (*Thuja plicata*) during the forest trip (Supplementary Tables 1 and 2). We detected several phytoncides such as α-pinene, limonene, and (1R)-(-)-Myrtenol in the outdoor forest fields and α-pinene, limonene, (1R)-(-)-Myrtenol, camphor, and β-caryophllene in the indoor wood house. Studies suggested that phytoncides may partially contribute to the enhanced activating NK cells during the forest trip [8–11]. Our study also demonstrated that most cardiovascular characteristics (age, male gender, SBP, and triglyceride, hs-CRP, fasting glucose levels, and smoking and tea drinking habits) affected NK cell percentage, thereby indicating the potentially confounding factors of NK cell measurements.

Furthermore, we detected significantly lower concentrations of gaseous air pollutants, such as NO, NO_2_, NO_x_, SO_2_, and CO, in the forest environment compared with the urban environment during the study period. Thus, the beneficial effects on immune function suggest a potential link between the better air quality in the forest environment and human health. Ambient air pollution can affect child and perinatal health on immune function by low-dose insults as compared with adult health [47–50]. Exposure to air pollutant toxicity may cause immunosuppression and result in increased expression of aberrant immune responses [47].

The strength of this study was the consistent detection of health effects in terms of increased percentage of NK cells in the observation study among workers living in forest environment and activating NK cells among middle-aged adults who went on the short-term forest trip. The results of air quality monitoring in Xitou forest are better than those in Taipei in this study and our previous study [21]. However, this study has several limitations. First, the differences in NK cell percentage between the two groups are associated with the cardiovascular characteristics of participants, such as hypertension status, hs-CRP levels, male, hyperglycemia, and being overweight. Thus, the results may overestimate the difference between two groups. However, controlling all confounding factors would result in over-adjustment. These factors may have attenuated the health effects of the forest environment on the percentage of NK cells. Second, although our study indicated the health effects of a forest trip on activating NK cells, we could not infer that the actual benefits were due to phytoncides in Xitou forest. The specific health effects of biogenic volatile organic compounds from tree leaves in forest environments were not investigated during this study, however the real measurements of phytoncides in the major tree of Xitou forest have been demonstrated in this study site [44]. Third, we did not compare the same study group in the urban trip with the group who went on the forest trip in terms of the percentage of NK cells and activating NK cells. Fourth, the sample size of the forest trip group is relatively small, which may cause the results to be overestimated or underestimated. Fifth, the small difference in the percentage of NK cells and activation NK cells cannot provide strong evidence that will allow us to infer the beneficial effects of exposure to a forest environment.

In conclusion, this study suggests that exposure to forest environments might enhance the immune response of NK cells and activating NK cells in humans. The health effects of a forest environment are important areas of research. Further studies are anticipated to understand the mechanisms that mediate the health effects of forest environments. Such studies should consider not only the phenomenological observations but also the potential influencing factors of NK cells and activating NK cells in humans.

## MATERIALS AND METHODS

### Study design and population

The study design aimed to determine the health effects on NK cells among staff members who live in a forest environment compared with those of who live in an urban environment. We recruited 90 staff members who live in the forest and 110 urban staff members who live in Taipei for an observational pilot study, which was conducted in the forest or urban environment for more than one year (Supplementary Figure 1). All participants provided informed consent before being subjected to cardiovascular health and biochemical examinations and NK cell measurements. The health examinations of forest participants were conducted from January 6 to 10, 2014 in Xitou, NTU Experimental Forest, Nantou County, and from January 14 to 17, 2014 for urban participants in Taipei.

To demonstrate the health effects of a forest trip on middle-aged subjects, we recruited 15 middle-aged volunteers who had been living in Taipei for more than one year in a small pilot study (Supplementary Figure 2). Subjects with clinical diabetes, major diseases, and documented cardiovascular diseases were excluded. All participants had to undergo cardiovascular health and biochemical examinations, and their NK cells had to be measured before the forest trip. A total of 11 participants with a mean age of 60.4 years were selected. To prevent interference by dietary habits, each subject had to be subjected to dietary control (limited dietary fat and calorie intake according to the National Cholesterol Education Program Step 1 diet) [51] for at least 10 days before leaving for Xitou forest.

These 11 subjects joined a five-day/four-night forest trip in Xitou Experimental Forest from January 6 to 10, 2014. During the forest trip, all participants had to maintain dietary control and walking exercise. On the first day, the participants walked for 1.5 hours (approximately 1.8 km) in the afternoon in a forest field and then stayed in a wooden house in the forest. In the next three days, the participants walked in two different forest fields for 1.5 hours in the morning and afternoon, respectively. On the fifth day, after their blood was drawn and a health examination was completed, all participants finished the forest trip and returned to Taipei. We took blood samples prior to the forest trip as a control sample on January 3. Overnight fasting blood was also sampled for each participant on the final day (January 10, 2014) of the forest trip and on the fourth day (January 14, 2014) after the forest trip. All blood samples were obtained at 9:00 a.m. on the study day and were placed in an ice/water box at 4°C. Assays were performed within four hours from the blood being drawn to measure the white blood cell counts, percentage of activating NK cells, and proportions of NK cells in peripheral blood. To prevent the potential bias of NK cells due to skin injury or bee bites during the forest trip, every participant had to record any skin injury or bee bites if they occurred.

This study was approved on the thirty-seventh meeting (January 30, 2013) of the Research Ethics Committee of the NTU Hospital. All participants provided their written informed consent before receiving a series of detailed examinations and questionnaires.

### Site descriptions of the forest and urban environments

The NTU Experimental Forest in Xitou and the interior office of a commercial building in Taipei served as the forest and urban environment sites, respectively. The Xitou Experimental Forest covers approximately 2,349 ha and is mainly a natural hardwood forest with some plantations that predominantly contain conifers. The annual rainfall was 2,590 mm between 1941 and 2010, where 80% of the rainfall occurred between May and September. The mean relative humidity and temperature from 2011 to 2015 were 88% and 18°C, respectively, according to the Xitou monitoring station of the NTU Experimental Forest. Our environmental monitoring site was located at an elevation of 1150 m near the meteorological station (23°40N 120°47E) of Xitou Experimental Forest. The commercial building was a financial building located near the Taipei main station in Taipei. Our study subjects worked for a financial securities corporation. Interior office air quality monitoring was conducted on the twenty-first floors of the commercial building. The mean relative humidity and temperature were 73% and 24°C, respectively, according to the Taipei monitoring station of the Central Weather Bureau [52].

### Exposure assessments

The instruments were set up in the Xitou Experimental Forest and in the interior office of the commercial building (indoor environment) in Taipei. The monitoring system was composed of a nitrogen oxide (NO_x_) analyzer (Model CLD 88YP, ECO Physics, Switzerland), a sulfur dioxide (SO_2_) analyzer (Model 43i-TLE, Thermo Scientific Inc., USA), a carbon monoxide (CO) analyzer (Model 48i-TLE, Thermo Scientific Inc., USA), an ozone (O_3_) analyzer (Model 49i, Thermo Scientific Inc., USA), and a temperature and relative humidity probe (Metone 083C, Met One Inc., Oregon, USA). Forest environment monitoring data were collected by the environmental monitoring system from January 6 to 10, 2014. The SO_2_, NO_x_, CO, O_3_, temperature, and relative humidity measurements were recorded daily every minute throughout the examination period.

The air conditioning in this building is a central control system. To obtain the indoor monitoring data of the urban environment, the same environmental monitoring system was set up in the commercial building to record daily data every minute from January 14 to 17, 2014. For each hour, air quality data were extracted from a monitoring data system and averaged to one result for each air pollutant. In addition, daily measurements of outdoor monitoring data of the urban environment from the Taipei air quality monitoring stations operated by the Taiwan Environmental Protection Administration were applied during the study period from January 14 to 17, 2014. The monitoring stations were fully automated and provided hourly readings of SO_2_ (Ecotech 9850B, Australia) and NO_x_ (Ecotech 9841B, Australia) by ultraviolet fluorescence, CO (Horiba APMA360, Japan) by non-dispersive infrared photometry, O_3_ (Ecotech 9810B, Australia) by ultraviolet photometry, temperature, and relative humidity (083C, Met One Inc., Oregon, USA).

For each participant, blood pressure was measured twice after at least 5 min of rest in a sitting position. The blood samples were obtained after overnight fasting for 10–14 hours via the antecubital vein for each participant. Low- and high-density lipoprotein cholesterol (LDL-C and HDL-C), plasma glucose and serum levels of cholesterol, and triglycerides were measured using an auto-analyzer (Toshiba, TBA-200FR; Toshiba, Tokyo, Japan). Serum high-sensitivity C-reactive protein (hs-CRP) was measured using chemiluminescent enzyme-labeled immunometric assay (Immulite C-Reactive Protein, Diagnostic Products Co., Los Angeles, USA).

### Blood sample preparation and flow cytometry analysis

The samples were prepared according to the method described by Jackson et al. 1986 [31]. Freshly isolated peripheral blood lymphocytes (PBLs) were stained with immunomodulatory agents by following combinations of directly labeled monoclonal antibodies (mAbs): CD3 piperidinin-chlorophyll conjugate (PerCP)/CD56 PE/CD69 fluorescein isothyocyanate (FITC) (Becton Dickinson, CA, USA) for 30 minutes at 4°C in the dark. After incubation cells were suspended with 8 mL of a 1/10 dilution FACS Lysing solution (BD Biosciences) in the dark for 20 min at room temperature. Cells were centrifuged at 1500 rpm for 5 minutes for removing the supernatant. Cells were then washed with PBS twice by centrifugation at 1500 rpm for 5 minutes. Finally, the supernatant was discarded and the cells suspended in 200 μL of PBS for flow cytometry analysis. NK cell level was defined as CD3^−^/CD56^+^ population. Activating NK cell was defined as CD3^−^/CD56^+^/CD69^+^ population. Surface marker expression was quantified on a FACSVerse flow cytometer (Becton Dickinson). A total of 20,000 gated events verified as lymphocytes according to their physical characteristics (forward scatter and side scatter) were collected per sample. The results were expressed as the percent of a cell subset using CellQUEST software.

### Statistical analyses

We compared the general characteristics and percentage of NK cells and activating NK cells in the forest and urban groups. The *t*-test was used to detect differences in the group means for the characteristic variables if they were continuous and normally distributed, whereas the chi-squared test was applied to categorical data. Continuous variables were expressed as the mean ± standard deviation, and binary variables were expressed as percentages.

The environmental conditions were compared based on the air quality and meteorological data collected in the forest and urban environments. The complete blood cell counts were also compared between the forest and urban groups. The percentages of NK cells and activating NK cells in the forest and urban groups were compared using the crude value and regression model adjusted value after adjusting for age and gender.

The percentages of NK cells and activating NK cells in the forest and urban groups were also compared through subgroup analyses, including age, gender, BMI, hypertension, hs-CRP, and fasting glucose. The percentages of NK cells and activating NK cells before and after a short-term forest trip were compared through a paired *t*-test to detect intra-individual differences among participants. All the statistical analyses were performed with SAS statistical software (version 9.2, SAS Institute Inc., Cary, NC, USA).

## SUPPLEMENTARY MATERIALS FIGURES AND TABLES


