# Supplementary material for: Health effects of a forest environment on natural killer cells in humans: an observational pilot study
Source: Oncotarget. 2018 Mar 27;9(23):16501–11. doi: 10.18632/oncotarget.24741 (PMC5893257; doi:10.18632/oncotarget.24741)
Supplement: Supplementary file 1 [file oncotarget-09-16501-s001.pdf]

Health effects of a forest environment on natural killer cells in humans: an observational pilot study

SUPPLEMENTARY MATERIALS

Supplementary Methods

Negative ion concentrations of outdoor air were measured every minute in forest and urban environments by using an air ion counter (COM-3600F, COM-system Co. Ltd, Japan). Negative ion concentrations in a forest environment were collected every morning for 3 hours from June 21 to 25, 2016. Negative ion concentrations were collected every morning for 4.5 hours in an urban environment from August 22 to 23, 2016. Negative ion concentrations in a wooden house made of Western red cedar were collected for a total of 24 hours from June 24 to 25, 2016.

Instruments based on a time-of-flight analyzer (MS-200, Kore Technology, Ely, Cambridgeshire, UK) were used to record data during field analysis of phytoncide in forest and urban environments. Phytoncide concentrations of outdoor air in a forest environment were measured for a total of 16 times from June 21 to 25, 2016. Phytoncide concentrations of outdoor air in an urban environment were measured for a total of 10 times from August 22 to 23, 2016. Phytoncide concentrations in a wooden house made of Western red cedar were measured for a total of four times from June 24 to 25, 2016.

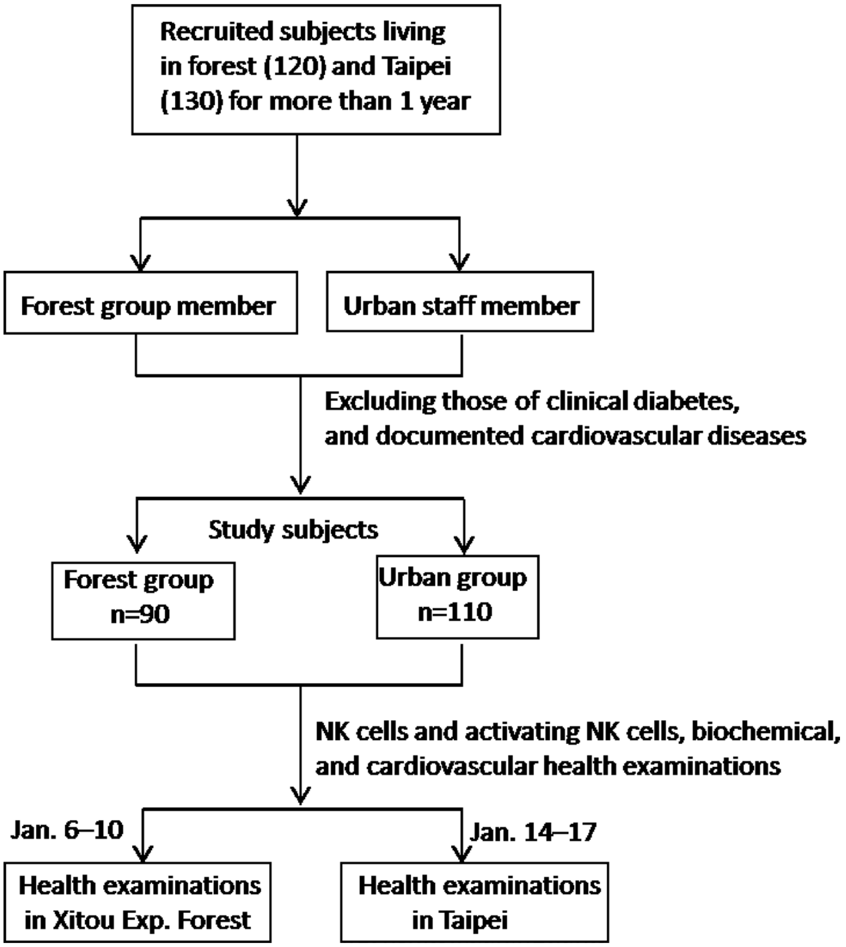

Supplementary Figure 1: Flowchart for the study design of subjects living in forest and urban environments.

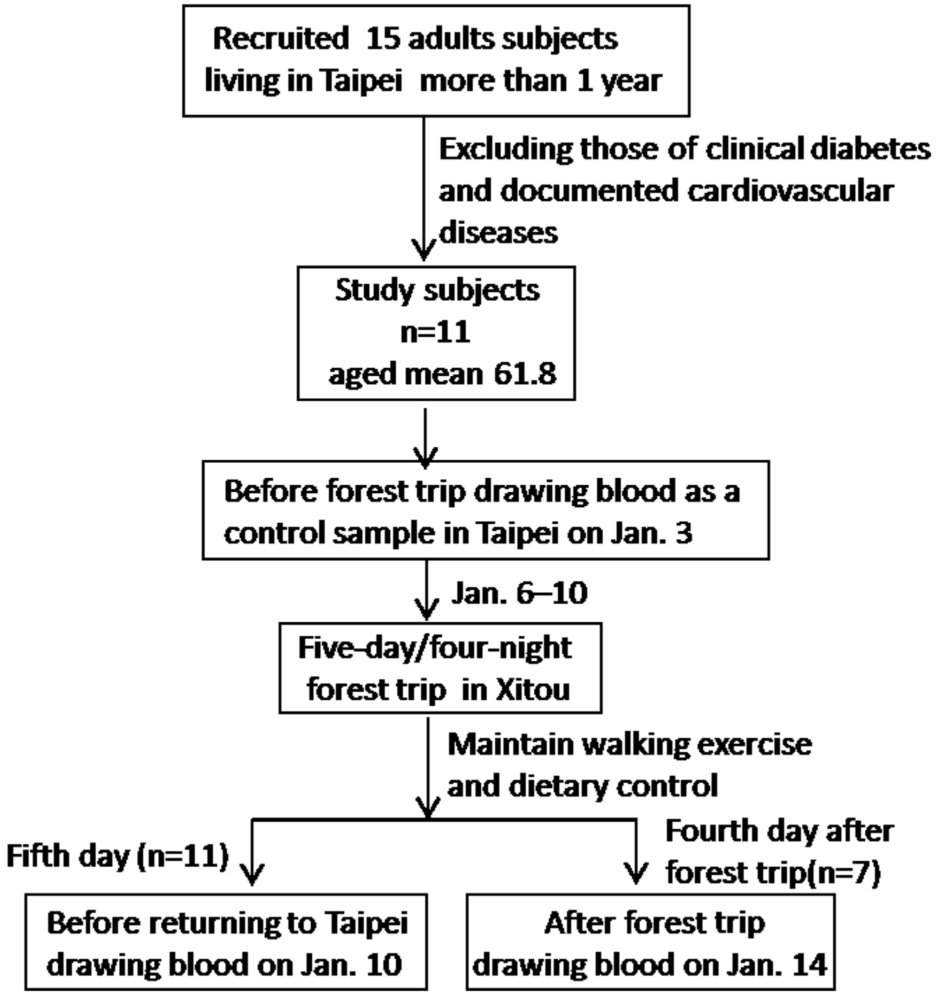

Supplementary Figure 2: Flowchart for the study design of the forest trip.

Supplementary Table 1: Outdoor air components in forest and urban environments

Air components	Unit	*N*	Forest	*N*	Urban
**Negative ion**	**counts/cm^3**	**900**	**502.2 ± 227.5**	**540**	**367.7 ± 153.0**
**Phytoncides**					
α-Pinene	ppb	16	11.8 ± 18.9	10	ND
Limonene	ppb	16	1.6 ± 1.8	10	ND
(1R)-(-)-Myrtenol	ppb	16	21.5 ± 25.6	10	ND

ND: not detectable.

Supplementary Table 2: Indoor air components in a wooden house made of Western red cedar

Air components	Unit	*N*	Forest
**Negative ion**	**counts/cm^3**	**1440**	**737.4 ± 424.4**
**Phytoncides**			
α-Pinene	ppb	4	11.8 ± 18.9
Limonene	ppb	4	366.5 ± 393.2
(1R)-(-)-Myrtenol	ppb	4	107.3 ± 88.1
Camphor	ppb	4	8.0 ± 6.9
β-Caryophllene	ppb	4	3182.5 ± 1929.6
